# Tumor-induced natural killer cell dysfunction is a rapid and reversible process uncoupled from the expression of immune checkpoints

**DOI:** 10.1126/sciadv.adn0164

**Published:** 2024-08-28

**Authors:** Kévin Pouxvielh, Marie Marotel, Annabelle Drouillard, Marine Villard, Marion Moreews, Anna Bossan, Mathilde Poiget, Liliane Khoryati, Sarah Benezech, Lucie Fallone, Sarah Hamada, Noémi Rousseaux, Louis Picq, Yamila Rocca, Aurore Berton, Marine Teixeira, Anne-Laure Mathieu, Michelle Ainouze, Uzma Hasan, Alain Fournier, Olivier Thaunat, Antoine Marçais, Thierry Walzer

**Affiliations:** ^1^ CIRI, Centre International de Recherche en Infectiologie, Univ Lyon, Inserm, U1111, Université Claude Bernard Lyon 1, CNRS, UMR5308, ENS de Lyon, F-69007 Lyon, France.; ^2^ Sanofi Oncology Research, Vitry-Sur-Seine, France.

## Abstract

Natural killer (NK) cells often become dysfunctional during tumor progression, but the molecular mechanisms underlying this phenotype remain unclear. To explore this phenomenon, we set up mouse lymphoma models activating or not activating NK cells. Both tumor types elicited type I interferon production, leading to the expression of a T cell exhaustion-like signature in NK cells, which included immune checkpoint proteins (ICPs). However, NK cell dysfunction occurred exclusively in the tumor model that triggered NK cell activation. Moreover, ICP-positive NK cells demonstrated heightened reactivity compared to negative ones. Furthermore, the onset of NK cell dysfunction was swift and temporally dissociated from ICPs induction, which occurred as a later event during tumor growth. Last, NK cell responsiveness was restored when stimulation was discontinued, and interleukin-15 had a positive impact on this reversion. Therefore, our data demonstrate that the reactivity of NK cells is dynamically controlled and that NK cell dysfunction is a reversible process uncoupled from the expression of ICPs.

## Teaser

Natural killer cell reactivity decreases rapidly upon activation by tumor cells, independently of immune checkpoints.

## INTRODUCTION

Natural killer (NK) cells are innate cytotoxic lymphocytes with important roles in antiviral immune responses (*1*) and in immunosurveillance (*2*) of cancers, especially hematological malignancies (*3*–*5*). NK cells display several effector functions, including granule-dependent cytotoxicity and secretion of inflammatory cytokines [tumor necrosis factor–α (TNF-α) and interferon-γ (IFN-γ)] and chemokines (CCL3 to CCL5) that are thought to have complementary roles during immune responses (*6*). Target cell recognition by NK cells is finely regulated through a wide range of activating (e.g., NKG2D, DNAM-I, NKp30, and NKp46) and inhibitory (e.g., KIRs and NKG2A) receptors. Mice deficient in activating NK cell receptors NKG2D, NKp46, and DNAM1 are more susceptible to different types of transplanted or spontaneous tumors (*7*–*9*). NK cell activation and effector functions are triggered when activating signals overcome inhibitory ones, the latter being provided upon recognition of major histocompatibility complex class I (MHC-I) molecules. Mechanistically, this involves opposing actions of kinases and phosphatases on common substrates (*10*). Tumor cells may lose MHC-I expression as a result of genomic instability combined with CD8 T cell–mediated editing (*11*), and MHC-I–negative cells are often strongly susceptible to NK cell–mediated lysis (*12*), a phenomenon known as missing-self recognition (*13*).

However, NK cells can become dysfunctional during cancer development not only in mouse models but also in humans. Different mechanisms could explain this dysfunction, including nutrient deficiencies related to the tumor microenvironment (*14*) as well as feedback mechanisms associated with chronic NK cell stimulation, which could predominate in MHC-I–deficient tumors (*15*, *16*). In such tumors, infiltrating NK cells acquire an anergic state manifesting as poor responsiveness to stimulation (*15*, *16*). NK cell dysfunction has been associated with either a decreased expression of activating NK cell receptors (*17*) or the transcriptional induction of inhibitory receptors from the immune checkpoint protein (ICP) family such as Lymphocyte Activation Gene 3 (Lag-3) (*18*), T cell Immunoreceptor with Ig and ITIM domains (TIGIT) (*19*, *20*), and T cell Immunoglobulin and Mucin domain-containing protein 3 (Tim-3) (*21*, *22*). In some studies (*23*) but not others (*24*), Programmed cell Death protein 1 (PD-1) expression has also been observed on dysfunctional NK cells, which could be the result of a trogocytosis process (*25*). However, if these changes in ICP expression are the cause, then the consequence or just correlates of dysfunction remain poorly understood. In many studies, cytokine treatments such as interleukin-21 (IL-21) (*23*), IL-12 (*18*), or IL-12/18 (*16*), either alone or in combination with ICP blockers (*14*, *18*), have been used to improve the antitumor function of NK cells. However, in most of these studies, it remains unclear if the beneficial effect of cytokines stemmed from priming NK cells that were not yet dysfunctional, from a genuine reinvigoration of dysfunctional cells, or from a bystander effect on other cells such as T cells. Along the same line, the positive effect of ICP blockers on NK cell function observed in a previous study may be indirect given that ICPs are largely shared among immune cell types (*26*).

The objective of this study was to establish an in vivo tumor model to investigate NK cell dysfunction, explore its association with ICPs, and evaluate its potential reversibility.

## RESULTS

### A mouse model for studying interactions between NK and tumor cells

**Fig. 1.**
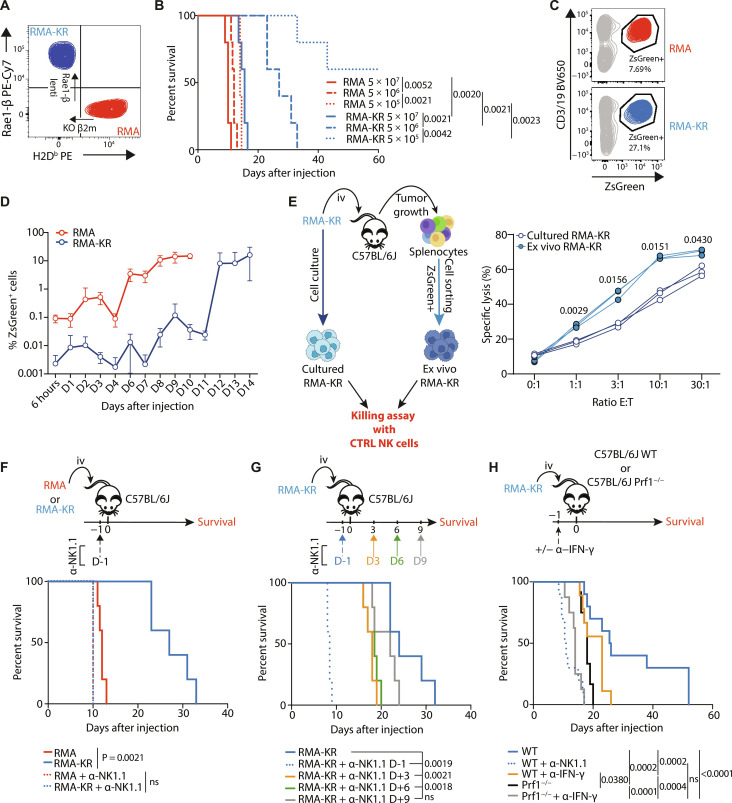
NK cells control of RMA-KR tumors. (**A**) Flow cytometry analysis of H2D^b^ and Rae1-β expression by RMA and RMA-KR cells. PE, phycoerythrin. (**B**) Survival analysis of mice injected intravenously (iv) with different numbers of RMA or RMA-KR cells, as indicated (*n* = 5 mice per group). Kaplan-Meier curves are shown and analyzed using the log-rank (Mantel-Cox) test (right). (**C**) Flow cytometry analysis of ZsGreen and CD3/19 (combined staining) in spleen cells from mice previously injected with tumor cells. (**D**) Kinetic analysis of the percentage of RMA or RMA-KR tumor cells among spleen cells in mice previously injected with tumor cells (total of 74 mice for RMA and 115 for RMA-KR). (**E**) RMA-KR cells were FACS-sorted from the spleen of C57BL/6J mice at late stages of tumor growth and used as targets in a cytotoxicity assay using resting NK cells as effectors; the original RMA-KR cultured line was used as the control (CTRL) (*n* = 3 mice per group). Two-way analysis of variance (ANOVA) followed by Sidak’s multiple comparison test. (**F**) Survival curve of mice previously depleted or not of NK cells by means of anti-NK1.1 injection [day −1 (D-1)] and injected with RMA or RMA-KR cells (day 0). The pictogram above the graph describes how the experiment was performed (*n* = 5 mice per group). (**G**) Survival curve of mice previously depleted or not of NK cells by means of anti-NK1.1 injection (timing as indicated) and injected with RMA or RMA-KR cells (day 0) (*n* = 5 mice per group). The pictogram above the graph describes how the experiment was performed. (**H**) RMA-KR cells were injected intravenously into *Prf1*^−/−^ mice or control mice previously treated or not at day −1 with anti–IFN-γ and the overall survival was followed (*n* = 8 to 12 mice per group). The pictogram above the graph describes how the experiment was performed. In (F) to (H), Kaplan-Meier curves are shown and analyzed using the log-rank (Mantel-Cox) test (right). ns, not significant.

To study the mechanisms of NK cell dysfunction, we developed a mouse model in which NK cells were highly activated by the tumor, given the established link between dysfunction and activation (*16*), and in which we could extract large numbers of NK cells. We modified the RMA lymphoma line by means of CRISPR-Cas9–mediated genome editing to knock out *B2m* expression. This resulted in a cell line devoid of surface MHC-I molecules H2D^b^, H2K^b^, and B2m expression (Fig. 1A and fig. S1A). *B2m*-deficient RMA cells were then transduced with a lentivirus expressing the ZsGreen fluorescent protein and the strong NKG2D ligand Rae1-β to obtain the RMA-*B2m* knockout (KO) Rae1-β^+^ line (thereafter called RMA-KR) (Fig. 1A and fig. S1A). RMA-KR cells were cloned by limiting dilution to prevent changes in their MHC-I/Rae1-β phenotype over time, and we verified that this phenotype was stable over culture passages (fig. S1A). As expected, RMA-KR cells were highly susceptible to NK cell–mediated killing (fig. S1B) and induced NK cell degranulation and production of IFN-γ (fig. S1C). RMA cells bearing only one of both modifications (RMA-*B2m*-KO or RMA-Rae1-β) displayed intermediate immunogenicity between parental RMA and RMA-KR cells. We chose to focus on the two extreme cell lines RMA and RMA-KR and injected intravenously different numbers of those in C57BL/6J mice then monitored mouse survival. As previously shown (*23*), RMA cells were very aggressive and led to a rapid death of mice, irrespective of the number of tumor cells injected (Fig. 1B). By contrast, mice receiving RMA-KR cells survived much longer, and this survival was inversely proportional to the number of tumor cells injected (Fig. 1B). Next, we analyzed RMA and RMA-KR tumor growth in the spleen, the main NK cell reservoir, taking advantage of ZsGreen expression by RMA and RMA-KR cells (Fig. 1C). RMA cells started growing in an exponential manner as soon as they were injected. By contrast, tumor growth was delayed in the RMA-KR model, with a lag phase followed by exponential growth (Fig. 1D). The latter phase was likely not due to tumor editing as (i) the expression of B2m and Rae1-β by RMA-KR cells was similar at early and late time points of tumor growth (fig. S1D) and (ii) RMA-KR cells sorted from mice at late stages of tumor growth remained highly susceptible to NK cell killing (Fig. 1E). In some experiments, we also monitored tumor growth in different organs. A visual inspection of the liver at late stages of tumor growth indicated the presence of large metastases (fig. S1E). A detailed flow cytometry analysis indicated that RMA-KR lymphoma cells preferentially infiltrated the liver, bone marrow, lungs, and spleen, and, to a lesser extent, the brain, lymph nodes, and blood (fig. S1F).

When we depleted NK cells by means of NK1.1 antibody injection prior to tumor cell injections, there was no longer a difference in survival between RMA- and RMA-KR–injected animals (Fig. 1F). Of note, NK cell depletion had little impact on the survival of RMA-injected mice (Fig. 1F), while T cell depletion did not influence mouse survival in any of the two models (fig. S1G). We then investigated the duration of tumor control by NK cells. To this aim, we depleted NK cells at different time points related to tumor injection in recipient mice, from day −1 up to day 9 and monitored mouse survival. The effect of NK cell depletion was more important when performed at day −1, showing that NK cell control of tumors occurred mainly at early stages. Yet, when this depletion was performed at day +3 and day +6, the effect on mouse survival remained significant, suggesting a sustained control of tumor growth by NK cells (Fig. 1G). We then asked what NK cell effector functions were required for the control of RMA-KR cells in vivo. To address this question, we injected RMA-KR cells into *Prf1^−/−^* mice that lack perforin or control mice that were treated with blocking anti–IFN-γ antibody or vehicle and monitored survival in the different mouse groups. Both perforin and IFN-γ were important for the control of RMA-KR cells, and the combined blockade of both components abrogated the protection mediated by NK cells, as shown by the near superposition of survival curves of wild-type (WT) mice depleted of NK cells and *Prf1^−/−^* mice that received anti–IFN-γ antibody (Fig. 1H).

Overall, our data show that NK cells control the early growth of RMA-KR lymphoma cells in a perforin- and IFN-γ–dependent manner. However, this control is temporary and the RMA-KR lymphomas eventually grow.

### IFNAR-dependent NK cell activation in both RMA and RMA-KR tumor models

**Fig. 2.**
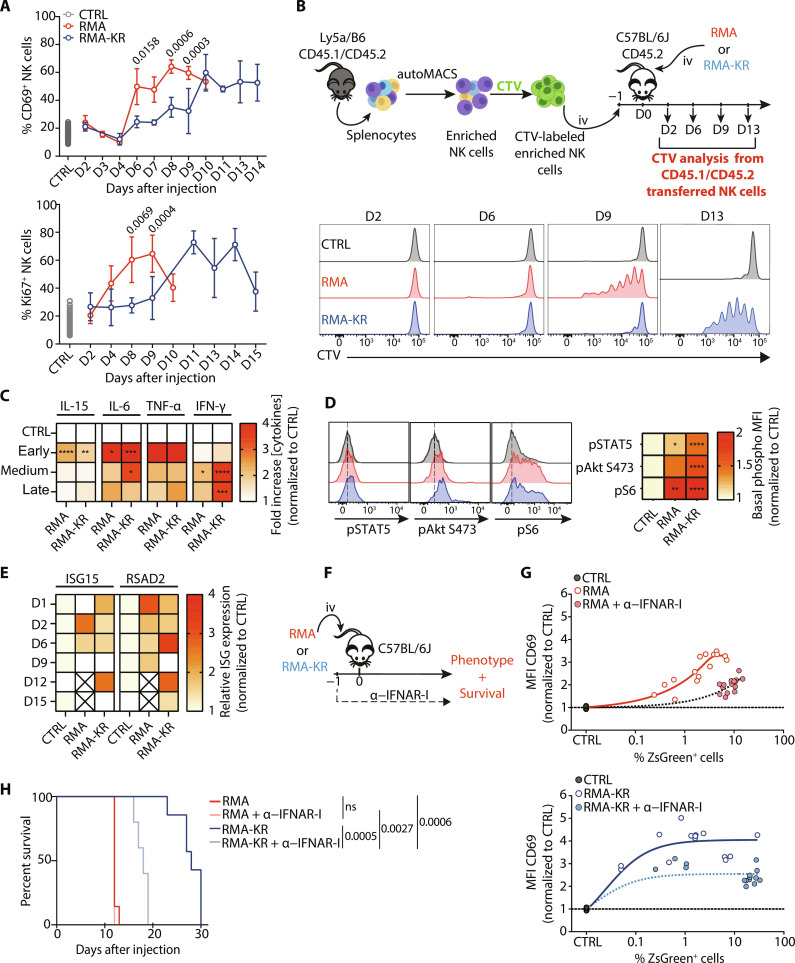
NK cells are activated by IFN-I and proliferate upon tumor growth. (**A**) The percentage of CD69^+^ or Ki67^+^ splenic NK cells was measured by flow cytometry following tumor injection [(top) *n* = 39 to 72 mice per group]. Graphs show means ± SD. The Mann-Whitney test was used for group comparisons. (**B**) NK cells purified from Ly5a x C57BL/6J mice were stained with CTV and transferred into C57BL/6J mice. A total of 5 × 10^7^ RMA or RMA-KR cells were then injected, and mice were euthanized at the indicated time points to monitor NK cell proliferation. The pictogram above the graphs describes the experiment. Representative CTV histograms are shown (*n* = 3 mice per group per time point). (**C**) ELISA measurement of cytokine levels in the spleen exudate at early (day 2), intermediate (day 4 for RMA and day 6 for RMA-KR), and late time points (day 9 for RMA and day 12 for RMA-KR). Results are expressed as heatmaps normalized to control (*n* = 8 to 42 mice per group). Two-way ANOVA followed by Sidak’s multiple comparison test was performed for group comparisons (*P < 0.05; **P < 0.01; ***P < 0.001; ****P < 0.0001). (**D**) Flow cytometry analysis of STAT5, Akt, and S6 phosphorylation in spleen NK cells from mice injected with RMA or RMA-KR cells 9 or 15 days before, respectively. Results are expressed as heatmaps normalized to control NK cells (*n* = 6 to 33 mice per group). A Kruskal-Wallis analysis followed by Dunn’s multiple comparison test was used for group comparisons (*P < 0.05; **P < 0.01; ****P < 0.0001). MFI, mean fluorescence intensity. (**E**) RT-qPCR analysis of ISG15 and RSAD2 expression in spleen cells from mice injected with RMA or RMA-KR cells at the indicated time points. Results are expressed as heatmaps, normalized to control NK cells (*n* = 12 to 18 mice per group). (**F** to **H**) Mice were treated or not with IFNAR blocking antibody before tumor injection as depicted in (F). (G) Kinetic analysis of CD69 expression in NK cells (*n* = 5 to 15 mice per group). The curves were fitted using a nonlinear model. (H) Kaplan-Meier graph of mouse survival (*n* = 5 to 7 mice per group), analyzed according to the log-rank (Mantel-Cox) test (right).

We then monitored the expression of activation markers CD69 and Ki67 during tumor growth in the spleen. Results were expressed as the percentage of NK cells positive for each marker over time (Fig. 2A) or relative to the percentage of tumors in the spleen (fig. S2A) so as to overlay results with RMA and RMA-KR cells on the same scale. NK cells were activated in the course of tumor growth in both RMA and RMA-KR models, as demonstrated by coordinated up-regulation of CD69 and Ki67 (Fig. 2A). In both models, NK cell activation not only was directly proportional to tumor load in the spleen but also occurred for lower tumor loads in the case of the RMA-KR tumor (fig. S2A). We then assessed NK cell proliferation during tumor growth. This was achieved by transferring CellTrace Violet (CTV)-labeled NK cells from donor Ly5a x C57BL/6J mice into C57BL/6J mice that were then injected with tumors. As shown in Fig. 2B, we observed NK cell proliferation in both models, but this proliferation became evident only at later stages of tumor growth (day 9 for RMA and days 12 and 13 for RMA-KR), long after NK cells had lost their ability to control the tumor. Furthermore, this proliferation did not lead to increased numbers of total NK cells as the number of mature NK cells dropped during tumor progression in the RMA-KR model (fig. S2, B and C).

Because NK cells have an activated phenotype in both RMA- and RMA-KR–bearing mice despite being directly stimulated only by RMA-KR cells, we suspected a role of cytokines in their activation. To address this question, we first monitored the concentration of different cytokines in the spleen exudate during tumor growth. We detected an early production of inflammatory cytokines IL-6, TNF-α, and IL-15 and a late production of IFN-γ in both tumor models, with a higher level of cytokines for RMA-KR tumors (Fig. 2C and fig. S2D). To determine if NK cells were exposed to IL-15, we measured the basal levels of phosphorylated signal transducer and activator of transcription 5 (STAT5), Akt, and S6 because these phosphorylation events are dependent on IL-15 engagement in NK cells (*27*). Results showed an increase in the phosphorylation level of these proteins in both models over time after tumor injection (Fig. 2D). IL-15 is known to prime NK cell antitumor function downstream of type I IFN (IFN-I) (*28*), and transplanted tumors induce Stimulator of Interferon Genes (STING)-dependent IFN-I production (*29*). Because IFN-I is difficult to detect in vivo, we monitored the expression of IFN-stimulated genes (ISGs) in splenocytes from mice injected with RMA or RMA-KR cells. Reverse transcription quantitative polymerase chain reaction (RT-qPCR) analyses showed an up-regulation of ISGs in spleen cells in both models (Fig. 2E), suggesting that IFN-I drives NK cell proliferation indirectly by inducing IL-15 expression. To test the role of IFN-I in both models, we then treated mice with an Interferon alpha/beta Receptor (IFNAR) blocking antibody before tumor injection (Fig. 2F). In both tumor models, IFNAR blockade delayed NK cell activation as measured by CD69 expression (Fig. 2G). Furthermore, blocking IFNAR accelerated the death of mice injected with RMA-KR cells but showed no impact on those injected with RMA cells (Fig. 2H). These results suggest that IFN-I primes the capacity of NK cells to kill immunogenic tumors, as shown previously in different MHC-I–negative tumor models (*30*).

Collectively, our data show that the injection of RMA or RMA-KR cells induces an IFN-I–dependent cytokine cascade that culminates in NK cell activation and proliferation, leading to tumor control in the RMA-KR model.

### Broad suppression of NK cell reactivity upon growth of the RMA-KR tumor

**Fig. 3.**
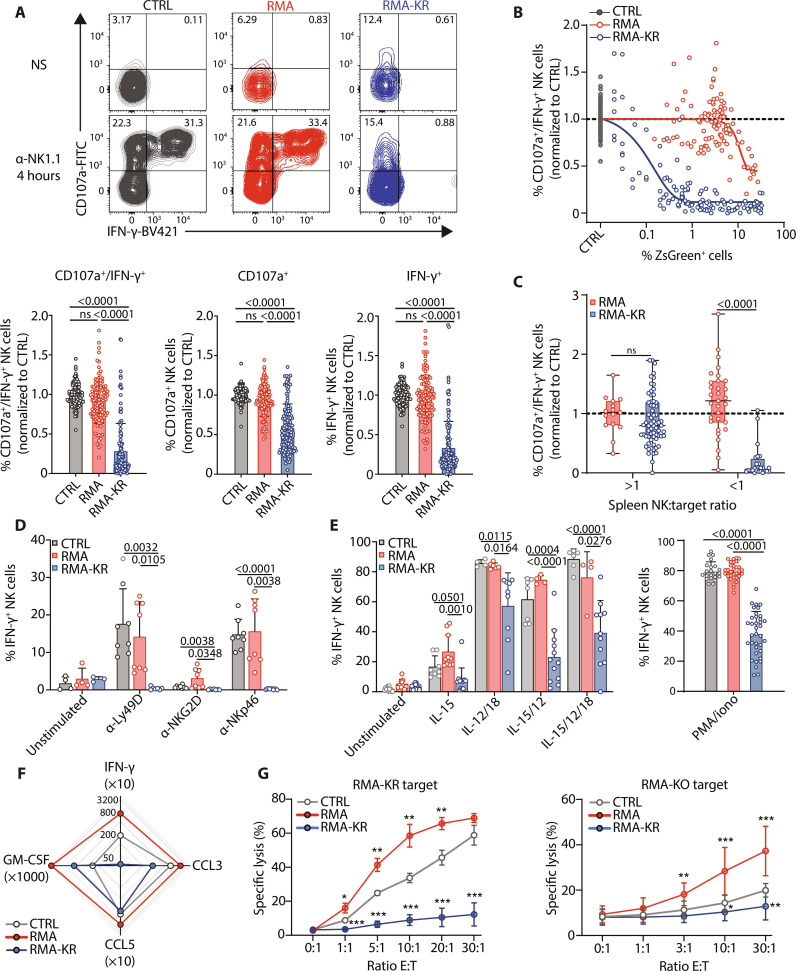
RMA-KR tumor progression induces broad NK cell dysfunction. Spleen NK cells from RMA- or RMA-KR–bearing mice were analyzed for different effector functions at different stages of tumor growth. (**A** to **C**) Spleen cells were stimulated for 4 hours with plate-bound NK1.1 antibody, and surface CD107a and intracellular IFN-γ were measured by flow cytometry. (A) (Top) Representative FACS plot of CD107/IFN-γ expression in spleen NK cells at late stages of tumor growth (*n* = 93 to 131 mice per group). NS, nonstimulated. (Bottom) Histogram analysis of means ± SD expression of CD107/IFN-γ. A Kruskal-Wallis analysis followed by Dunn’s multiple comparison test was used for group comparisons. (B) Analysis of the percentage of CD107a^+^ IFN-γ^+^ NK cells after NK1.1 stimulation relative to the percentage of tumor cells in the spleen (*n* = 90 to 215 mice per group). The curves were fitted using a nonlinear model. (C) Analysis of the percentage of CD107a^+^ IFN-γ^+^ NK cells after NK1.1 stimulation relative to the ratio between spleen NK cells and tumor cells (*n* = 15 to 66 mice per group). Graphs show means ± SD and a Mann-Whitney analysis. (**D** and **E**) The percentage of IFN-γ^+^ NK cells was measured in response to stimulation by anti-NKG2D, anti-Ly49D, or anti-NKp46 antibodies (*n* = 7 to 8 mice per group) (D) or different cytokines (*n* = 4 to 21 mice per group) or PMA/ionomycin (*n* = 22 to 36 mice per group) (E). Graphs show means ± SD and two-way ANOVA analysis followed by Tukey’s multiple comparison test [(D), left, and (E)] or Kruskal-Wallis analysis followed by Dunn’s multiple comparison test [(D), right]. (**F**) Levels of different cytokines produced by NK cells from the indicated mice (late stages of tumor growth) after stimulation with NK1.1 antibody (*n* = 5 to 7 mice per group). (**G**) Cytotoxicity assay of NK cells from the indicated conditions (late stages of tumor growth) against RMA-KR or RMA-KO (*n* = 4 to 15 mice per group) cell lines. Graphs show means ± SD and a two-way ANOVA analysis followed by Tukey’s multiple comparison test (*P < 0.05; **P < 0.01; ***P < 0.001).

To test if NK cells became dysfunctional in the RMA-KR model, we restimulated total splenocytes from the different models with a cross-linking anti-NK1.1 antibody and measured IFN-γ secretion and degranulation. Results in Fig. 3A show that, in mice bearing well-developed RMA lymphomas, NK cells retain a reactivity close to that of controls. In contrast, in the case of RMA-KR lymphomas, NK cells lost their reactivity in terms of both degranulation and IFN-γ production (Fig. 3A). This loss of function was progressive in the RMA-KR model and directly proportional to the tumor mass (Fig. 3B). In particular, when the overall ratio of NK cells to lymphoma cells was below 1, the function of NK cells significantly declined (Fig. 3C). Of note, there was also a partial decrease in NK cell reactivity in the RMA model but at much higher tumor loads. We calculated that a 50% decrease in NK cell reactivity required only 0.07% RMA-KR cells in the spleen and >200 times more RMA cells (19.52%; fig. S3A). We also observed that NK cells from RMA-KR mice were broadly dysfunctional because they did not react upon stimulation by cross-linking antibodies directed at activating receptors Ly49D, NKG2D, or NKp46; cytokines IL-15, IL-12, and IL-18; and, even to some extent, phorbol 12-myristate 13-acetate (PMA)/ionomycin (Fig. 3, D and E). Moreover, their dysfunction not only was not limited to the loss of CD107 and IFN-γ release but also resulted in lower amounts of granulocyte-macrophage colony-stimulating factor (GM-CSF), CCL3, and CCL5 compared to activated NK cells from RMA-bearing mice as measured by enzyme-linked immunosorbent assay (ELISA) (Fig. 3F). Combining NK1.1 stimulation with different cytokines improved the reactivity of not only dysfunctional NK cells but also control NK cells so that the difference in reactivity was maintained between both cell states under these conditions (fig. S3B). Dysfunction also affected NK cells in other organs from RMA-KR–bearing mice, including blood and liver NK cells (fig. S3, C and D). We also monitored the ex vivo capacity of sorted spleen NK cells from mice bearing similar loads of RMA and RMA-KR to kill RMA-KR or RMA-KO cells. As shown in Fig. 3G, NK cells from RMA-bearing animals had an increased killing capacity compared to control NK cells, confirming that they were primed by cytokines (*31*), while NK cells from RMA-KR–bearing mice were completely ineffective under the same conditions. Dysfunction in the RMA-KR model was not due to the conversion of NK cells into ILC1s (*32*) because they did not express CD49a and remained Eomes positive (fig. S3E). Moreover, it was not linked to maturation as all maturation stages defined by CD11b and CD27 were dysfunctional (fig. S3F).

Together, these results show that persistent stimulation of NK cells by RMA-KR tumors suppresses their reactivity through activating receptors and associated effector functions, regardless of maturation stage or anatomical localization.

### Dysfunctional NK cells from RMA-KR–bearing mice are phenotypically and transcriptionally similar to NK cells from RMA-bearing mice but express more ICPs

**Fig. 4.**
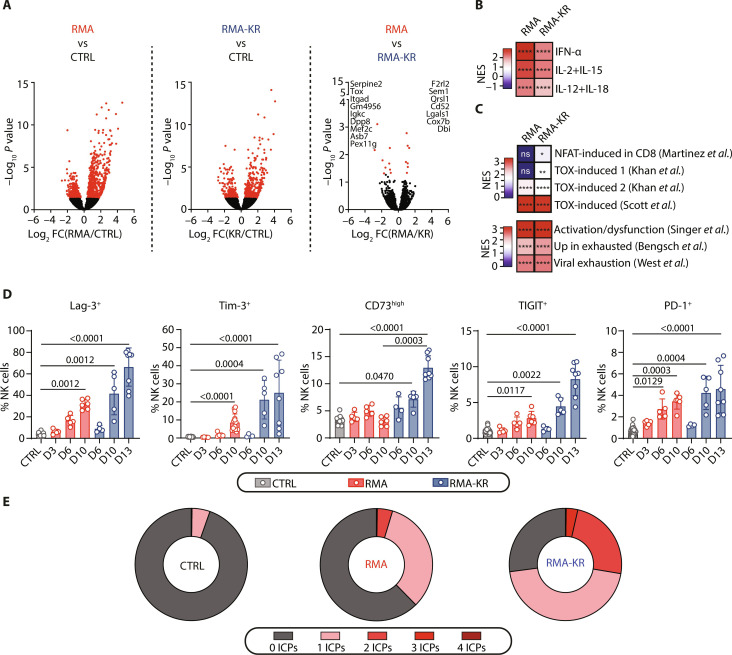
NK cell dysfunction is not induced through transcriptional mechanisms. (**A** to **C**) NK cells from RMA- or RMA-KR–bearing mice at late stages of tumor growth or from control mice were sorted by flow cytometry and subjected to RNA-seq analysis. (A) Volcano plots showing differential gene expression [fold change (FC) > 2 and adj. *P* value < 0.05] between RMA and control, RMA-KR and control, or RMA and RMA-KR conditions. (B) GSEA of the indicated gene modules [NK cell activation by cytokines (*33*)] in NK cells from RMA- or RMA-KR–bearing mice compared to controls. NES, Normalized Enrichment Scores. (C) GSEA of the indicated T cell exhaustion gene modules in NK cells from RMA- or RMA-KR–bearing mice compared to controls [(A) to (C) *n* = 3 mice per group]. For (B) to (C), the false discovery rate *q* values were determined compared to control (*P < 0.05; **P < 0.01; ****P < 0.0001). (**D**) Flow cytometry analysis of the percentage of NK cells expressing the indicated ICPs at the indicated time after tumor injection (*n* = 15 to 24 mice per group). Graphs show means ± SD, and a Kruskal-Wallis analysis followed by Dunn’s multiple comparison test was performed (*P* values are presented). (**E**) Coexpression of different ICPs (*n* = 6 to 14 mice per group).

To understand the causes of NK cell dysfunction, we then performed a total RNA sequencing (RNA-seq) analysis of NK cells from RMA-KR–bearing mice, in comparison with NK cells from control or RMA-bearing mice at late stages of tumor growth. Hundreds of genes were differentially expressed between control NK cells and NK cells from tumor-bearing mice (Fig. 4A and table S1). This included multiple genes involved in the regulation of the cell cycle (tables S1 and S2), confirming our previous analysis of NK cell proliferation. A gene set enrichment analysis (GSEA) returned a substantial enrichment for cytokine-mediated activation of NK cells (*33*), confirming that cytokines produced during tumor growth shape NK cell gene expression (Fig. 4B). Moreover, several ICPs such as Lag-3 and TIGIT were up-regulated in NK cells from tumor-bearing mice (table S1). However, when we compared NK cells from mice bearing RMA and RMA-KR tumors, only 16 genes were differentially expressed. The transcription factor Tox was one of them and was slightly higher in dysfunctional NK cells (Fig. 4A and fig. S4A). Because Tox is a master regulator of T cell exhaustion, we next analyzed the expression of gene modules previously associated with T cell exhaustion (*34*–*39*) in NK cells from tumor-bearing mice. We found a significant enrichment of these signatures in NK cells from both tumor models compared to controls, sometimes more significantly in NK cells from the RMA-KR model (Fig. 4C). Because Tox is known to induce the expression of ICPs, we then measured the expression of several ICPs by flow cytometry. Results in Fig. 4D and fig. S4B show that several ICPs are induced on NK cells in both models. Lag-3, Tim-3, and CD73 showed the most significant induction, with higher expression levels observed in mice carrying RMA-KR tumors compared to those carrying RMA tumors. TIGIT and PD-1 were expressed at low levels, the latter expression being confirmed using several antibodies (fig. S4C). Moreover, ICPs were coexpressed on NK cells from tumor-bearing mice, especially in the RMA-KR model (Fig. 4E). Thus, dysfunctional NK cells in RMA-KR tumors are phenotypically and transcriptionally similar to NK cells in RMA tumors but express more ICPs.

### NK cell dysfunction is uncoupled from ICP expression

**Fig. 5.**
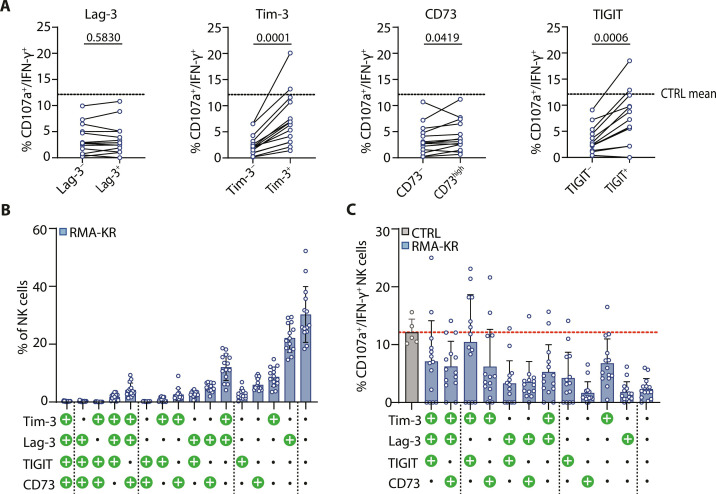
ICP-positive NK cells are more reactive than ICP-negative NK cells. Spleen cells were stimulated for 4 hours with plate-bound cross-linking NK1.1 antibody and stained for surface CD107a, various ICPs, and intracellular IFN-γ before analysis by flow cytometry. (**A**) Coexpression of CD107a and IFN-γ relative to the expression of the indicated ICPs in NK cells from RMA-KR–bearing mice is shown (*n* = 14). (**B**) Proportion of NK cells from RMA-KR–bearing mice expressing indicated different ICP combinations. (**C**) Coexpression of CD107a and IFN-γ relative to the combined expression of different ICPs from RMA-KR–bearing mice compared to control NK cells (*n* = 5 to 14 mice per group).

To determine if NK cell dysfunction in mice with RMA-KR tumors was due to the higher expression of ICPs or to their coexpression, we subsequently evaluated the responsiveness of ICP-positive and ICP-negative NK cells within the RMA-KR tumor-bearing mice. Our initial focus was to analyze the impact of each individual ICP. As shown in Fig. 5A, ICP-expressing NK cells were, at least, as responsive, if not more so, than ICP-negative NK cells, regardless of the ICP or the stimulation mode (fig. S5A). A small fraction of NK cells coexpressed two to three ICPs (Fig. 5B), but these cells were more reactive than ICP-negative NK cells (Fig. 5C). Of note, the single expression of some ICPs like Tim-3 or TIGIT was more positively correlated with NK cell reactivity than that of Lag-3 and CD73. Moreover, the individual effect of each of these ICPs added up when they were coexpressed because Tim-3^+^ TIGIT^+^ NK cells were as reactive as control NK cells (Fig. 5C).

To further test the role of ICPs in NK cell dysfunction, we then used blocking ICP antibodies. Ex vivo blockade of ICPs in cultures of total splenocytes from RMA-KR–bearing mice did not restore NK cell responsiveness, even when several ICPs were targeted (fig. S5B). Likewise, in vivo blockade of Tim-3 or Lag-3 did not improve the overall mouse survival of mice injected with RMA-KR cells or functionality of NK cells from RMA-KR–bearing mice (fig. S5C).

Collectively, these data show that NK cell dysfunction is uncoupled from ICP expression in the tumor models we used.

### The expression of ICPs and functional paralysis occur at distinct time intervals, each triggered by different signals

**Fig. 6.**
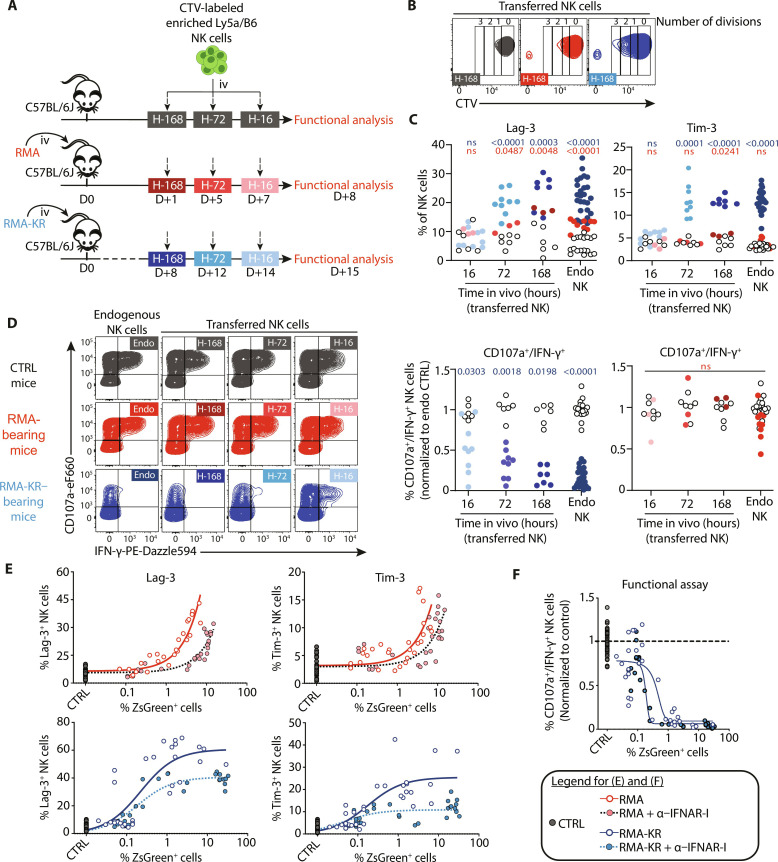
Dynamics of NK cell dysfunction, ICP induction, and proliferation in tumor-bearing mice. (**A** to **D**) NK cells purified from Ly5a x C57BL/6J mice were stained with CTV and injected 16, 72, or 168 hours before end stage of tumor growth in RMA- or RMA-KR–bearing mice, as outlined in (A); control mice not injected with tumors were also included (*N* = 3 to 9 per condition). At the indicated time point (day 8 after RMA injection or day 15 after RMA-KR injection), spleen cells from transferred mice were analyzed by flow cytometry. (B) FACS plots of CTV fluorescence in transferred NK cells after 168 hours in recipient mice. (C) Percent of Lag-3^+^ and Tim-3^+^ cells among endogenous or transferred NK cells at the indicated time point (blue, RMA-KR recipient mice; red, RMA recipient mice; white, control recipient mice). (D) Reactivity of transferred NK cells to NK1.1-mediated stimulation, as determined by coexpression of CD107a and IFN-γ. Representative FACS plots of CD107a/IFN-γ expression in endogenous or transferred NK cells are shown for the different conditions on the left, and graphs of CD107a^+^/IFN-γ^+^ NK cells in individual mice (normalized to control NK cells) are shown on the right. Each point represents a single mouse, and a Kruskal-Wallis analysis followed by Dunn’s multiple comparison test was performed. (**E** and **F**) Mice were treated intraperitoneally with IFNAR blocking antibody (200 μg) at day −1 and injected intravenously with RMA or RMA-KR cells at day 0. The percentage of NK cells expressing Lag-3 or Tim-3 (E) was monitored at different stages of tumor growth. NK cell reactivity (F) expressed as the percentage of CD107a^+^ IFN-γ^+^ NK cells in response to NK1.1 antibody and relative to control NK cells was also measured relative to the percentage of tumor cells in the spleen (*n* = 27 to 33 mice per group). The curves in (E) to (F) were fitted using a nonlinear model.

Next, we aimed to compare the time required for the induction of ICPs and the dysfunction of NK cells in tumor-bearing mice. To achieve this, we conducted an adoptive transfer of CTV-labeled Ly5a x C57BL/6J NK cells into C57BL/6J mice with tumors 16 hours, 3 days (72 hours), or 7 days (168 hours) before euthanizing the mice, which coincided with the terminal stage of tumor growth. CTV labeling also enabled monitoring NK cell proliferation under the different conditions. Control mice were also transferred with labeled NK cells to ensure that the transfer did not alter them (Fig. 6A). NK cell proliferation was detectable only for NK cells that had spent the longest time in tumor-bearing mice (7 days; Fig. 6B and fig. S6A). In contrast, the expression of ICPs was induced more rapidly in transferred cells as the expression of Lag-3 and Tim-3 was detectable starting from 3 days of presence in RMA-KR tumor-bearing mice (Fig. 6C). Last, NK cell dysfunction in transferred cells was the swiftest event in the mice as 16 hours in RMA-KR tumor-bearing mice were sufficient to render the NK cells dysfunctional, in terms of both degranulation and IFN-γ production. However, this dysfunction was slightly less severe than that of endogenous NK cells or those transferred and left in the mice for a longer period (Fig. 6D and fig. S6B). No changes in NK cells were detected in control cells, while the transfer into RMA-bearing mice induced only ICPs and no dysfunction (Fig. 6, C and D).

Because ICPs were induced on NK cells in both tumor models, we suspected a contribution of inflammatory cytokines in their induction, as shown in NK or T cells in other studies (*40*–*42*). We therefore measured ICP expression in NK cells from mice bearing RMA or RMA-KR and treated or not with an antibody blocking IFNAR. As the results in Fig. 6E show, IFNAR blockade delayed the induction of ICPs on NK cells in both tumor models. We then measured the ability of NK cells from tumor-bearing mice treated or not with the IFNAR blocking antibody to respond to ex vivo stimulation. IFNAR treatment had a rather negative effect on NK cell function in both RMA- and RMA-KR–bearing mice despite efficient suppression of ICP induction (Fig. 6F and fig. S6C), thus showing that the factors leading to NK cell dysfunction and ICP induction can be separated.

Together, these results show that ICPs and functional paralysis are triggered by different signals, and they manifest at separate time intervals.

### NK cell dysfunction is reversible upon termination of activating NK cell receptor engagement and is improved by cytokine treatment

**Fig. 7.**
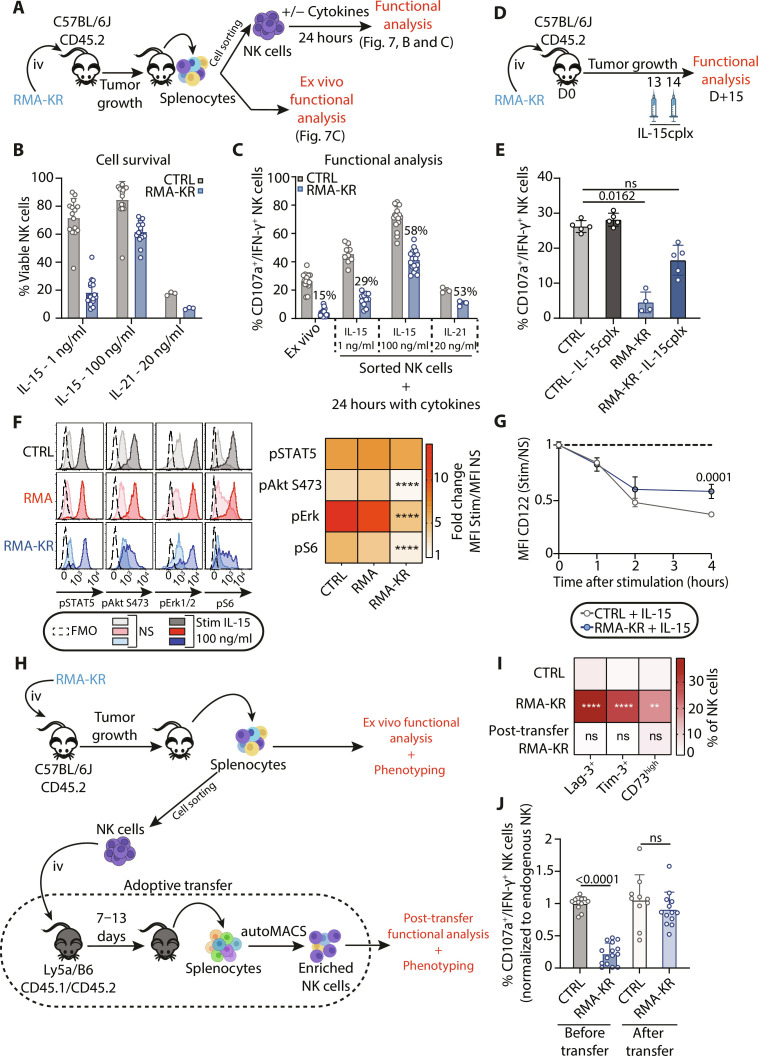
NK cell dysfunction is reversible upon discontinuation of stimulation or upon cytokine treatment. (**A** to **C**) NK cells were sorted from RMA-KR–bearing mice at late stages of tumor growth and then treated with cytokines before analysis. (A) Outline of the experiment. (B) FACS analysis of NK cell viability (*n* = 3 to 18 mice per group). (C) Reactivity of NK cells to NK1.1-mediated stimulation, as determined by coexpression of CD107a/IFN-γ. “Ex vivo” indicates NK cell reactivity before sorting and cytokine treatment (*n* = 3 to 18 mice per group). (**D** and **E**) Control and RMA-KR–bearing mice were injected twice with IL-15cplx as outlined in (D). (E) Reactivity of NK cells from the indicated groups to NK1.1-mediated stimulation (*N* = 5 per group). A Kruskal-Wallis analysis followed by Dunn’s multiple comparison test was used for statistical analysis. (**F** and **G**) Spleen cells from control or RMA- or RMA-KR–bearing mice at late stages of tumor growth were stimulated for 15 min (F) or different times (G) under the indicated conditions. (F) FACS analysis of different phosphoproteins (*n* = 6 to 33 mice per group). The left panels show the representative staining, and the heatmap (right) shows fold changes compared to nonstimulated NK cells (statistical analysis: two-way ANOVA followed by Tukey’s multiple comparison test, ****P < 0.0001). FMO, Fluorescence Minus One. (G) FACS analysis of CD122 surface expression (*n* = 3 to 8 mice per group). Statistical analysis: two-way ANOVA followed by Sidak’s multiple comparison test. (**H** to **J**) NK cells sorted from RMA-KR–bearing mice at late stages of tumor growth or control mice were transferred into Ly5a x C57BL/6J mice. They were analyzed before and after transfer. (H) Outline of the experiment. (I) FACS analysis of the percentage of ICP^+^ NK cells, expressed as a heatmap (*n* = 3 to 12 mice per group). **P < 0.01; ****P < 0.0001. (J) Coexpression of CD107a/IFN-γ in NK cells after 4-hour stimulation with NK1.1 antibody before and after transfer under the indicated conditions (*n* = 10 to 15 mice per group). Two-way ANOVA (I) or Kruskal-Wallis (J) analysis followed by Dunnett’s multiple comparison test were performed.

If NK cell dysfunction is a reversible state remains unknown, this question being of central importance for immunotherapy. To address it, we sorted dysfunctional NK cells from RMA-KR–bearing mice and cultured them in vitro in the presence of IL-15 or IL-21 because the latter has been suggested to rescue dysfunctional NK cells (Fig. 7A) (*23*). Low IL-15 concentrations allowed the survival of control but not dysfunctional NK cells, suggesting a reduced capacity of dysfunctional NK cells to signal through the IL-15 receptor (Fig. 7B). High IL-15 concentrations allowed the survival of all NK cell types and improved the reactivity of dysfunctional NK cells to NK1.1-mediated stimulation, but a difference with control NK cells persisted, suggesting that other signals were necessary to fully revert dysfunction (Fig. 7C). IL-21 did not allow NK cell survival but somewhat improved the reactivity of the surviving cells (Fig. 7, B and C). We then wanted to determine if treatment with IL-15 in vivo could improve NK cell function in mice bearing RMA-KR tumors. We administered two doses of the IL-15/IL-15Ra complex (IL-15cplx) (*43*) or phosphate-buffered saline (PBS) to the mice bearing RMA-KR tumors according to the protocol outlined in Fig. 7D, and NK cell function was tested the day after the second injection, which corresponded to end-stage tumor growth. As shown in the results of Fig. 7E, administration of the IL-15/IL15Ra complex significantly enhances the reactivity of dysfunctional NK cells. The reduced response of dysfunctional NK cells to IL-15 (Fig. 7C) is not related to STAT5, which is normally phosphorylated in response to this cytokine, but rather to the phosphorylation of Akt, Erk, and S6 (Fig. 7F), suggesting a downstream mammalian Target Of Rapamycin Complex 1 (mTORC1) activation defect of the IL-15 receptor. The β chain of the receptor (CD122) is less efficiently internalized in dysfunctional NK cells compared to controls (Fig. 7G).

The reduction in mTOR activity in dysfunctional NK cells echoes what we had previously observed for uneducated NK cells (*44*). This observation led us to question the educated or uneducated nature of dysfunctional NK cells. To test this, we repeated the experiment from Fig. 7 (A to C), but this time we analyzed the expression of major educating receptors in C57BL/6J mice, namely, Ly49C and NKG2A, after 24 hours of culture in IL-15. The results presented in fig. S7 (A and B) show that the fraction of educated NK cells is similar in dysfunctional NK cells compared to controls, at both low and high doses of IL-15. Furthermore, treatment of NK cells with a high concentration of IL-15 has the same effect in educated and uneducated NK cells, indicating that NK cell dysfunction in a tumor environment is independent of education.

Next, we wanted to determine if stopping in vivo stimulation would allow dysfunctional NK cells to regain their reactivity. To test this point, we adoptively transferred dysfunctional NK cells from RMA-KR–bearing or control mice into untouched C57BL/6J mice and monitored their phenotype and functions 7 days after transfer (Fig. 7H). As shown in Fig. 7 (I and J), dysfunctional NK cells lost the expression of several immune checkpoints (Lag-3, CD73, and Tim-3) previously expressed, and they also recovered full reactivity when stimulated through cytokines or activating receptors. This was unlikely due to the selection of a small functional population because the frequency of NK cells recovered from the RMA-KR condition was in the same order of magnitude as that of the control condition (fig. S7C).

Collectively, these results show that NK cell dysfunction is a reversible state in NK cells upon discontinuation of the activating signal and that dysfunctional NK cell reactivity can be rapidly improved upon cytokine treatment.

## DISCUSSION

Here, we used an in vivo model to study NK cell dysfunction based on a lymphoma cell line modified to control its susceptibility to NK cell killing. The advantages of this model compared to previous models of NK cell dysfunction are multiple. First, the RMA condition represents a control condition where NK cells ignore the tumor and thus where gene expression changes in NK cells are only induced via bystander stimulation. This is essential because most transcriptional changes in NK cells in our study were independent of tumor recognition by NK cells and therefore not causative of dysfunction, a point that was overlooked in many previous studies. Second, in our model, the tumor grows in the spleen, a major NK cell reservoir, which allows the supply of a great number of dysfunctional NK cells. This is a major improvement over previous studies that used solid tumors (*16*, *18*, *19*, *23*) that are poorly infiltrated with NK cells, which limits the number of dysfunctional cells that can be analyzed. Other studies have used in vitro models of NK cell dysfunction to circumvent this limitation, activating NK cells with cross-linking antibodies against NK cell receptors (*45*, *46*) or with tumor cells, but if such conditions are relevant to in vivo NK cell dysfunction is unknown. Third, our fluorescent tumor models allow monitoring NK cell–tumor interactions throughout tumor growth by flow cytometry and not only at end stages like in the case of solid tumors that cannot be analyzed before they reach a palpable size.

Our data show that activation and dysfunction are tightly linked in NK cells. The RMA tumor, which is not recognized by NK cells, does not induce substantial NK cell dysfunction, even at high tumor loads, confirming previous studies (*16*, *23*). Moreover, our data show that activation and dysfunction are staggered in time during tumor growth. NK cell depletion experiments revealed that tumor control was limited to the early stages (days 0 to 6 in our model), while dysfunction occurred at later time points (starting at days 7 to 9), when the number of tumor cells became equivalent to that of NK cells (1:1 ratios), suggesting a causal link between NK cell dysfunction and tumor growth.

Multiple studies have documented the induction of ICPs on tumor-infiltrating NK cells in mouse models (*16*, *18*, *19*, *23*), this expression being increased if tumors were MHC-I deficient (*16*, *23*) or if mice were treated with cytokines (*18*). Treatment with cytokines alone was also reported to be sufficient to induce ICPs (*47*). Here, we show that both tumor and IFN-I contribute to ICP induction, confirming data in T cells where IFN-I was identified as a primary signal of ICP induction (*40*). In RMA-KR–bearing mice, we found that ICP-positive NK cells were less dysfunctional than ICP-negative ones, and this contradicts a previous paper using solid tumors (*23*). However, in our study, we used a hematological tumor growing in the spleen rather than a solid tumor, where ICP-positive NK cells could be the only ones in contact with tumor cells. This distinction may introduce bias into the interpretation. Moreover, as detailed above, the study of NK cell dysfunction in solid tumors is only possible when the tumor is palpable, a stage that could follow NK cell dysfunction and where additional suppressive effects of the tumor not related to dysfunction may occur. Our study suggests that Tim-3^+^ and TIGIT^+^ NK cells are less dysfunctional than NK cells not expressing these ICPs. Inhibitory effects of ICPs could mitigate NK cell activation and therefore prevent dysfunction. This observation resonates with recent findings in the Chimeric Antigenic Receptor–T cells (CAR-T)/NK cell field, where studies have demonstrated that reducing the number of Chimeric Antigenic Receptor (CAR) activating modules (*48*) or combining CAR with inhibitory receptors (*49*) has a beneficial antitumor effect by mitigating CAR cell exhaustion.

Our data also show that lymphoma growth induced a transcriptional signature in NK cells similar to that of exhausted T cells, including multiple exhaustion-associated transcription factors such as Tox. Similarly, in a recent study, we demonstrated that dysfunctional human NK cells in individuals chronically infected with HBV (hepatitis B virus) exhibited a transcriptional signature similar to that of exhausted T cells induced by chronic viral infections, characterized by the expression of the exhaustion marker TOX (*26*). However, this Tox-associated transcriptional signature was neither necessary nor sufficient for NK cell dysfunction as shown by the similar induction of this signature in RMA- and RMA-KR–bearing mice and by the strong dysfunction occurring in the absence of ICP induction in RMA-KR–bearing mice treated with IFNAR antibodies. Similarly, in exhausted T cells, Tox deficiency prevents ICP expression but does not restore their reactivity (*27*–*29*), showing that the Tox-induced transcriptional program and ICPs do not explain functional exhaustion.

We have also studied the dynamics of changes induced during the antitumor response in NK cells. Analogous to what has been recently described in T cells (*50*), our data clearly show that loss of function is an event that occurs rapidly (within 16 hours) when transferring NK cells into tumor-bearing RMA-KR mice. This event is temporally disconnected from the induction of checkpoints or proliferation, which occurs later at 3 days and at least 7 days after transfer into the mice, respectively. Adding to this observation that dysfunctional NK cells do not have a specific transcriptional signature, our data suggest that the mechanism of dysfunction is linked to proximal changes in signaling mechanisms, which may be similar to what has been observed during NK cell education. These changes are likely induced after a period of sustained cytotoxicity, as suggested by the importance of perforin expression in the control of RMA-KR tumors. In vitro, it has been shown that human NK cells have limited efficacy in serial killing of target cells (*51*). Proximal changes may involve mTOR because a common feature of uneducated (*44*) and dysfunctional NK cells (this report) is a decreased mTOR activity.

Our data show that NK cell dysfunction is reversible upon discontinuation of the activating signal. Dysfunctional NK cells transferred into tumor-free mice rapidly recovered reactivity. This suggests that therapies aiming at reinvigorating dysfunctional NK cells should paradoxically target activating modules and, ideally, modules that would not interfere too much with NK cell reactivity (*34*). We found that cytokine treatments in vitro and in vivo had a positive effect on dysfunctional NK cells. This observation was in agreement with previous studies showing a positive impact of cytokine treatments on the function of dysfunctional NK cells (*16*, *21*, *23*). This effect may be related to the ability of several cytokines such as IL-15 or IL-18 to promote mTOR activation (*27*), the latter being a central checkpoint for NK cell reactivity (*44*). However, chronic stimulation by cytokines can also lead to NK cell dysfunction (*52*, *53*), which involves, in part, suppressors of cytokine signaling such as Cis (*54*) or Socs1 (*33*), thus suggesting that cytokines are not a reliable therapy to reinvigorate NK cells in a durable manner. More studies are needed to address this question.

In conclusion, our data demonstrate that NK cell dysfunction is a rapidly occurring event following activation by tumor cells, which happens prior to the induction of ICPs, whose expression preserves residual reactivity of NK cells. If contact with the tumor is halted, then dysfunctional NK cells can quickly recover their reactivity, providing insights into the design of antitumor therapies based on NK cells.

## MATERIALS AND METHODS

### Study design

This study was designed to investigate the mechanisms involved in NK cell dysfunction under tumor conditions in a mouse model. In each experiment, two tumor conditions were used (RMA, nonactivating condition; RMA-KR, activating condition) as well as tumor-free control mice. Mechanistically, the role of transcription and immune checkpoints in dysfunction was tested. The number of mice used in each experiment was not power-calculated, given the large number of questions asked. The project was validated by the local and national ethics committees. In each experiment, an end point based on a set of criteria was used, beyond which the mice were euthanized. In each experiment with tumor-bearing mice, data from mice in which tumors had not grown were excluded. No other exclusions were made. No randomization was performed, with the exception of experiments in which animal survival was monitored and for which the person in charge of monitoring did not know the treatment used or the tumor injected into each mouse. For each experiment, the number of animals used per condition was specified in the figure legends, as was the statistical test used to compare experimental conditions.

### Mice and in vivo procedures

All experimental procedures were approved by the local ethics committee CECCAPP according to the French recommendations in the guide for ethical evaluation of experiments using laboratory animals and the European guidelines 86/609/CEE. WT C57BL/6J mice were purchased from Charles River Laboratories (L’Arbresle). Ly5a heterozygotes mice and *Prf1^−/−^* were bred in our animal house. Female mice (7 to 9 weeks old) were used unless specified.

For intravenous injection, tumor cells were resuspended in 150 μl of Hanks’ balanced salt solution (Gibco) and 5 × 10^5^, 5 × 10^6^, or 5 × 10^7^ cells were injected in the tail vein. For functional analysis, mice were injected with 5 × 10^7^ tumor cells, compared to 5 × 10^6^ for survival analysis. For depletion experiments, monoclonal antibodies (mAbs) were purchased from BioXcell. To deplete NK cells, mice were injected intraperitoneally with 200 μg of PK136 on day −1 or on days 3, 6, and 9 relative to tumoral cell injection. To deplete T cells, mice were injected intraperitoneally with 100 μg of 53.5.8 clone (anti-CD8β) and 100 μg of GK1.5 (anti-CD4) on day −1 and every 5 days thereafter. Control mice were injected intraperitoneally with 200 μg of polyclonal anti-rat immunoglobulin G on day −1. Specific depletions were confirmed by cell staining using antibody clones different from the ones used for depletions. To block IFN-γ, mice were injected intraperitoneally with 200 μg of XMG1.2 on day −1. To block IFNAR-I, mice were injected intraperitoneally with 200 μg of MAR1-5A3 on day −1 and every 4 days thereafter. To block Lag-3, mice were injected intraperitoneally with 200 μg of C9B7W on day +3 (for RMA-bearing mice) or day +6 (for RMA-KR–bearing mice) and every 4 days thereafter. To block Tim-3, RMA-KR–bearing mice were injected with 200 μg of RMT3-23 on day +11 and every 4 days. For the IL-15/IL-15Rα complex (IL-15cplx) treatments, mice were injected intraperitoneally with 4 μg of IL-15cplx (*43*) twice within a 2-day interval.

### Culture and modification of the RMA cell line

All cell lines were cultured in RPMI 1640 medium (Invitrogen Life Technologies) supplemented with 10% fetal calf serum, 2 mM l-glutamine, 10 mM penicillin/streptomycin (HCL Technologies), 1 mM sodium pyruvate (PAA Laboratories), 20 mM Hepes (Gibco), and nonessential amino acids (Gibco). Cell cultures were maintained at 37°C in an incubator with a humidified atmosphere of 5% CO_2_/95% air and used for in vivo injection after three passages.

#### Gene editing

A single guide RNA targeting the first exon of the mouse *B2m* gene (sequence: AGTCGTCAGCATGGCTCGCT) was cloned into the pX458 plasmid (Addgene), which also contains the Cas9 gene. Then, 0.2 × 10^6^ parental RMA cells were transfected by electroporation using the Neon transfection system (Thermo Fisher Scientific; 1 pulse/50 ms/1080 V). Transfection efficiency was analyzed by green fluorescent protein (GFP) expression 24 hours after transfection. Cells were sorted for GFP expression 48 hours after transfection with the FACSAria II instrument. Rae1-β gene was subcloned from the MR220341 plasmid (Origene) into the pRRLSIN-MND-IRES2-ZsGreen-WPRE lentiviral vector (p297, Vect’UB vectorology platform, Bordeaux). After amplification, RMA or MHC-I–deficient RMA cells were transduced with the empty p297 lentivirus or containing Rae1-β at a multiplicity of infection of 50 and incubated for 24 hours. Forty-eight hours after transduction, cells were sorted for the expression of ZsGreen and subcloned.

### Flow cytometry analysis

A total of 1.5 × 10^6^ spleen cells were stained for 30 min at 4°C with the appropriate mAbs detailed in table S3. Intracellular staining for Ki67 and transcription factors was performed with the Foxp3 Fixation/Permeabilization buffer kit (eBioscience). Intracellular staining for cytokines and nontranscription factors proteins was performed with the Cytofix/Cytoperm kit (BD Biosciences). Intracellular staining of phosphorylated proteins was performed with Lyse/Fix and Perm III buffers (BD Biosciences). Phosphorylated proteins were stained for 1 hour at 4°C. Flow cytometry analysis was performed on a FACS Fortessa (BD Biosciences) or a Cytek Aurora (Cytek). Fluorescence Minus One controls were used to set the gates, and data were analyzed with the FlowJo 10.9.0 software (BD Biosciences).

### NK cell purification and sorting

#### Negative depletion

Spleen cell suspensions were incubated at 250 million/ml with biotinylated mAbs against CD3 (145-2C11, BioLegend), CD5 (53-7.3, BioLegend), Ly-6G (RB6-8C5, eBioscience), CD24 (M1/69, BioLegend), CD4 (GK1.5, BioLegend), CD8 (53-6.7, BioLegend), CD19 (1D3, eBioscience), and TER-119 (ter119, BioLegend), followed by incubation with anti-biotin microbeads (Miltenyi) for 15 min at 4°C for both steps. Then, splenocytes were washed and enriched by magnetic separation using autoMACS (Miltenyi) to obtain a cellular suspension containing 30 to 80% of NK cells.

#### Cell sorting

Cell suspensions were stained for 30 min at 4°C using the following antibodies: CD3 fluorescein isothiocyanate (FITC) (145-2C11, BioLegend), CD5 FITC (53-7.3, eBioscience), CD19 FITC (1D3, BD Biosciences), T cell receptor (TCR) α/β FITC (H57-597, BioLegend), TCR γ/δ FITC (GL3, BD Biosciences), NKp46 allophycocyanin (APC) (29A1.4, BioLegend), and NK1.1 APC (PK136, BD Biosciences) and a viability dye LIVE/DEAD fixable Near-IR (Invitrogen). Then, NK cells (APC^+^ FITC/Near-IR^−^) were sorted on an Aria II (BD Biosciences) to obtain 98 to 100% of purity.

### Cell culture and stimulation

To measure CD107a degranulation and IFN-γ expression, cell suspensions were stimulated for 4 hours on antibody-coated plates using anti-NK1.1 (PK136, BioXcell), anti -NKG2D (Mi-6, eBioscience), anti-Ly49D (4E5, BD Biosciences), or anti-NKp46 (29A1.4, home-made) at the indicated concentrations in Immulon 2 HB plates (Corning) or with PMA (50 ng/ml) and ionomycin (1 μM) and/or with recombinant mouse cytokines (PeproTech): IL-12 (25 ng/ml), IL-18 (5 ng/ml), or IL-15 (100 ng/ml), or cocultured with indicated target cell lines at a 5:1 target/effector ratio in the presence of GolgiStop (BD Biosciences) and anti-CD107a. The percentage of NK cells positive for CD107a and IFN-γ was then measured by flow cytometry.

For phospho-flow analysis, 3 × 10^6^ splenocytes were stimulated with IL-15 (100 ng/ml) for 15 min before fixation with Lyse/Fix. Cells were then permeabilized with Perm III buffer and stained. For the kinetic measurement of CD122 internalization, 1.5 × 10^6^ splenocytes were stimulated with IL-15 (100 ng/ml) for the indicated times and the surface level of CD122 was measured by flow cytometry.

In some experiments, total splenocytes were cultured for 24 hours with IL-15 (1 ng/ml) with or without blocking antibodies against Lag-3 and Tim-3 (C9B7W, RMT3-23; 10 μg/ml) and with or without CPI-444, an inhibitor of adenosine receptor A2AR. In other experiments, dysfunctional NK cells were fluorescence-activated cell sorting (FACS)–sorted then cultured for 24 hours with cytokines [IL-15 (1 or 100 ng/ml) or IL-21 (20 ng/ml)].

### In vitro cytotoxic assay

Sorted NK cells were cocultured for 4 hours with the indicated cell lines at different effector-to-target (E/T) ratios in 96-well V-bottom plates. The number of targets cells was set to 5000 cells per well (RMA or RMA-Rae1-β or RMA-KO or RMA-KR or RMA-KO-NanoLuc or RMA-KR-NanoLuc) and the final volume at 180 μl. The plates were spun down 2 min at 1200 rpm prior to the 4-hour coculture.

To quantify the death of target cells by flow cytometry, RMA or RMA-Rae1-β or RMA-KO or RMA-KR tumor cells were previously labeled with CTV dye at 2 μM in PBS (Thermo Fisher Scientific). After 4 hours, cells were collected, and dead cells were labeled using the LIVE/DEAD fixable Near-IR stain reagent from Invitrogen following the manufacturer’s recommendations. Cells were analyzed by flow cytometry, and the percentage of dead tumor cells was determined by gating on CTV^+^ cells.

To quantify the death of RMA-KO-NanoLuc or RMA-KR-NanoLuc, 50 μl of coculture supernatants was collected after 4 hours of coculture and transferred into white, flat-bottom 96-well plates. The NanoLuc activity was measured by adding 50 μl of the Nano-Glo Luciferase Assay System from Promega. The bioluminescence was measured for 0.1 s with TECAN Infinite 200 PRO. The maximum of bioluminescence (100% of dead target cells) was measured by collecting 50 μl of supernatants from target cells previously lysed with 180 μl of NP-40 lysing buffer.

### Assessment of NK cell proliferation and function upon transfer in lymphoma-bearing mice

NK cells from the spleen of Ly5a x C57BL/6J mice were first enriched by negative depletion and then stained with 2 μM Cell Trace Violet (Thermo Fisher Scientific) for 15 min at 37°C. The percentage of NK cells was calculated, and 1 × 10^6^ NK cells were injected in the ophthalmic venous sinus of control or RMA- or RMA-KR–bearing mice. Upon recipient mice euthanasia, splenic and liver NK cells were enriched by negative depletion. Proliferation was then monitored by measurement of CTV fluorescence using flow cytometry. NK cell functionality was measured in the same samples as exposed above. Transferred NK cells were distinguished from endogenous NK cells based on CD45 alleles (CD45.2 versus CD45.1-CD45.2).

### Measurement of spleen cytokine levels by ELISA

Spleens from control or RMA- or RMA-KR–bearing mice were collected at different time points and dissociated in 1 ml of complete medium. The cell suspension was then centrifuged, and the supernatant was collected. The level of different cytokines in the supernatant was measured by ELISA using the following kits: Mouse IFN-γ ELISA MAX Deluxe Set (BioLegend), Mouse IL-6 DuoSet (R&D Systems), Mouse IL-15 DuoSet (R&D Systems), and Mouse TNFα DuoSet (R&D Systems).

### ISG expression by RT-qPCR

One million splenocytes were lysed in TRIzol (Thermo Fisher Scientific). Total RNA was then extracted using the Direct-zol RNA microprep kit (Ozyme). The extracted RNA concentration was determined using the QuantiFluor RNA system (Promega). The level of ISGs was monitored by semiquantitative RT-PCR (StepOnePlus Real-Time PCR System, Thermo Fisher Scientific) using the following primers: ISG-15-R (CACGGACACCAGGAAATCGT; Sigma-Aldrich), ISG-15-F (AAGCAGCCAGAAGCAGACTC; Sigma-Aldrich), RSAD2-R (CTCAATTAGGAGGCACTGGAA; Sigma-Aldrich), RSAD2-F (GTGGACGAAGACATGAATGAAC; Sigma-Aldrich), GAPDH-R (TGTCATCATACTTGGCAGGTTTCT), and GAPDH-F (GCATGGCCTTCCGTGTTC). The results were normalized to glyceraldehyde-3-phosphate dehydrogenase (GAPDH).

### Analysis of cytokine production by in vitro stimulated NK cells by CBA

FACS-sorted NK cells were stimulated using plate-bound anti-NK1.1 (PK136, BioXcell) for 24 hours at 37°C. The culture supernatant was collected, and the amount of IFN-γ, CCL3, CCL5, and GM-CSF was measured by flow cytometry using Cytometric Bead Array (CBA; BD Biosciences) according to the manufacturer’s instructions.

### RNA sequencing

#### Sequencing

Total RNA was purified using the Direct-zol RNA microprep kit (Ozyme) according to the manufacturer’s instructions and was quantified using the QuantiFluor RNA system (Promega). RNA-seq libraries were generated using mRNAseq library Prep Kit V2 (LEXOGEN). Tagged library quality was checked on D1000 screen tape and analyzed on TapeStation 4200 (Agilent). Sequencing was performed by the GenomEast platform, a member of the “France Génomique” consortium (ANR-10-INBS-0009), on an Illumina HiSeq 4000 sequencing machine (read length, 1 x 50 nt).

#### Analysis

Reads were processed using an in-house RNA-seq pipeline of the GenomEast facility. Briefly, raw data were preprocessed using cutadapt 1.10 to remove the adaptor and low-quality sequences (Phred quality score below 20). Reads shorter than 40 bp were removed for further analysis. Remaining reads were mapped to mouse ribosomal RNA (rRNA) sequences using bowtie 2.2.8, and reads mapped to rRNA sequences were discarded for further analysis. Remaining reads were aligned to the mm10 assembly of the mouse genome with STAR 2.5.3a. Gene quantification was performed with htseq-count 0.6.1p1 using the “union” mode and Ensembl 96 annotations. Differential gene expression analysis between groups of samples was performed using the method implemented in the Bioconductor R package DESeq2 1.16.1, with the following nondefault options: betaPrior = TRUE and alpha = 0.05. *P* values were adjusted for multiple testing using the Benjamini and Hochberg method.

### Adoptive transfer of NK cells to assess functional recovery

NK cells from control or RMA-KR–bearing mice were FACS-sorted and adoptively transferred into Ly5a x C57BL/6J mice via the retro-orbital sinus. After 7 to 13 days, NK cells were purified from recipient mice and their phenotype and functionality were assessed. Transferred NK cells were distinguished from recipient NK cells based on CD45 alleles (CD45.2 versus CD45.1-CD45.2).

### Statistical analysis

Statistical analyses were performed using Prism 6 (GraphPad Software). Different parametric or nonparametric tests were used, as appropriate and as indicated in the figure legends.
