# Supplementary material for: Tumor-induced natural killer cell dysfunction is a rapid and reversible process uncoupled from the expression of immune checkpoints
Source: Sci Adv. 2024 Aug 28;10(35):eadn0164. doi: 10.1126/sciadv.adn0164 (PMC11352832; doi:10.1126/sciadv.adn0164)
Supplement: Supplementary file 1 — Figs. S1 to S7 Legends for tables S1 to S3 [file sciadv.adn0164_sm.pdf]

Science Advances
AAAS

Supplementary Materials for

Tumor-induced natural killer cell dysfunction is a rapid and reversible process uncoupled from the expression of immune checkpoints

Kévin Pouxvielh *et al.*

Corresponding author: Antoine Marçais, antoine.marcais@inserm.fr; Thierry Walzer, thierry.walzer@inserm.fr

Sci. Adv. **10**, eadn0164 (2024)
DOI: 10.1126/sciadv.adn0164

The PDF file includes:

Figs. S1 to S7
Legends for tables S1 to S3

Other Supplementary Material for this manuscript includes the following:

Tables S1 to S3

Figure S1.

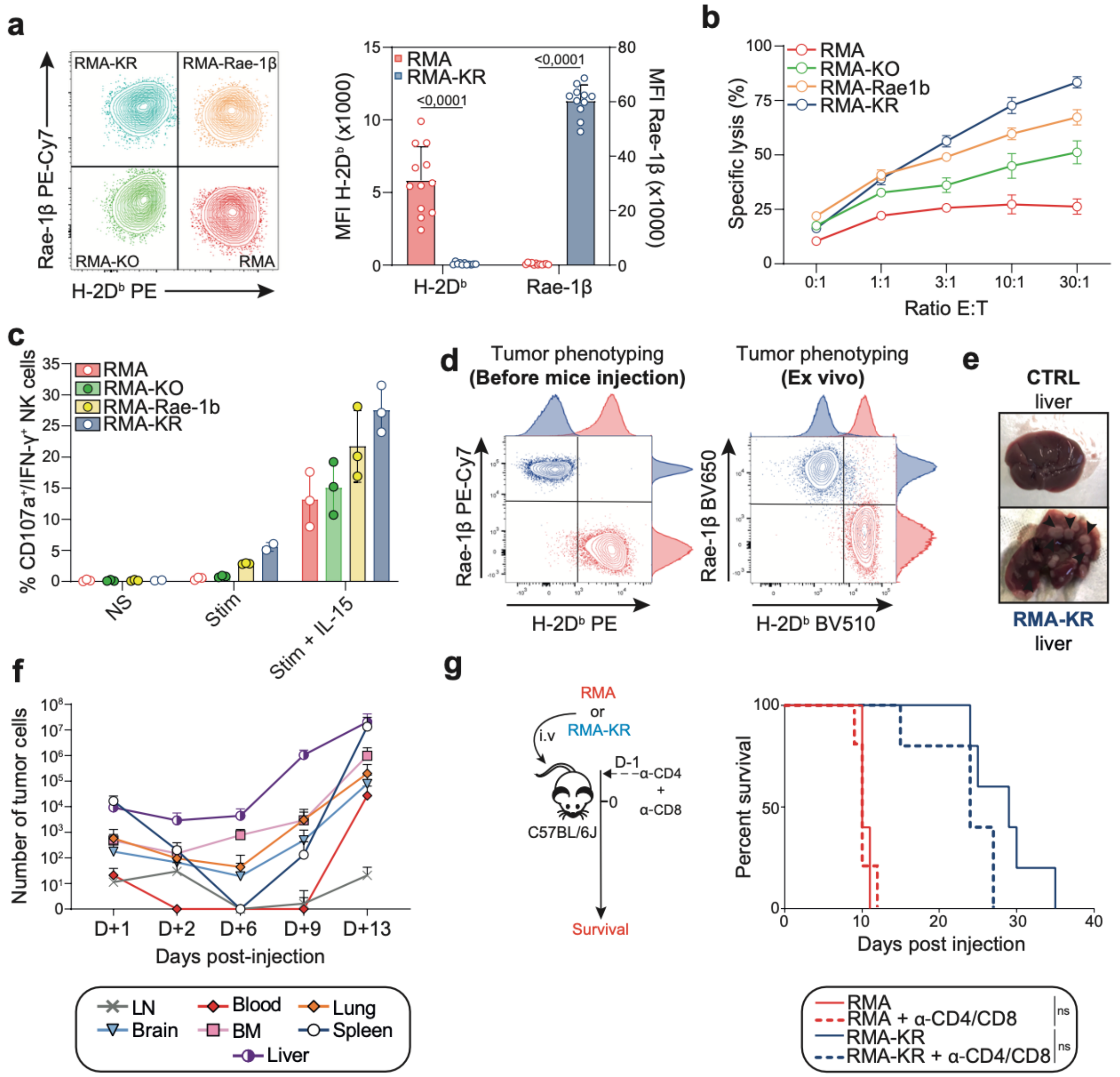

Figure S1. **Modifications of the RMA line and characterization of the tumor model**

(A) Flow cytometry expression of H-2D^b and Rae-1β expression in modified RMA lines. Left: density plot showing H-2D^b and Rae-1β expression in the indicated lines. Right: Mean fluorescence intensity of H-2D^b and Rae-1β staining measured at different cell passages. Graph show means ± SD and a Mann-Whitney analysis was performed (p-value are presented).

(B) Cytotoxicity assay of resting spleen NK cells against indicated cell lines (n=3 mice/group).

(C) Flow cytometry analysis of surface CD107 and intracellular IFN-γ expression upon 4-h stimulation with the indicated cell lines (n=3 mice/group).

(D) Flow cytometry analysis of H2Db & Rae1β expression by RMA-KR cells before injection (left panel) or isolated from the spleen of C57BL/6 mice at late stages of tumor growth (right panel).

(E) Pictures of livers from C57BL/6 mice at late stages of tumor growth.

(F) Flow cytometry analysis of the number of RMA-KR cells in different organs of C57BL/6 mice at the indicated stages of tumor growth (n=5 mice/group).

(G) Survival curve of mice previously depleted or not of CD4 and CD8 T cells by means of anti-CD4 and anti-CD8 injection (D-1) and injected with RMA or RMA-KR cells (D0) (n=5 mice/group). The pictogram at the left of the graph describes how the experiment was performed. Kaplan-Meier analyses according to the log-rank (Mantel-Cox) test were presented (right).

Figure S2.

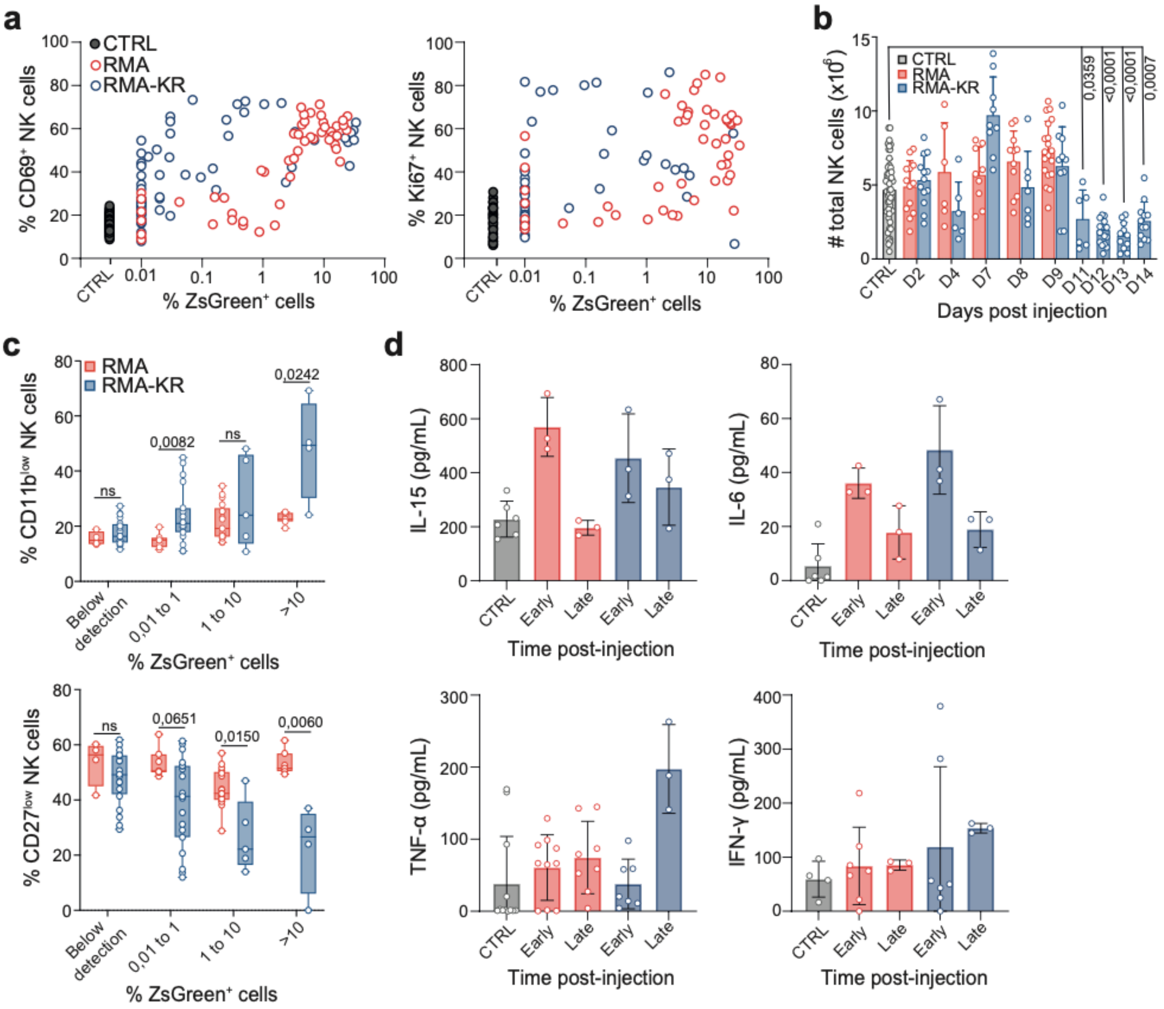

Figure S2: Maturation and number of NK cells during tumor growth

(A) The percentage of $CD69^{+}$ and $Ki67^{+}$ splenic NK cells were measured by flow cytometry over time after tumor injection and was expressed relative to the percentage of tumor cells in both RMA and RMA-KR models (n=48-72 mice/group).

(B-C) Flow cytometry analysis of spleen NK cells in C57BL/6 mice injected with RMA or RMA-KR cells. (B) Evolution of NK cell number during tumor growth (n=51-90). (C) Evolution of NK cell maturation, as defined by CD11b and CD27 expression during tumor growth (n=35-48 mice/group). For (B-C) graphs show means ± SD and Mann-Whitney analysis was performed (p-value are presented).

(D) Measurement of cytokine concentrations in the spleen exudate by Elisa, at different timepoints after tumor injection: early (Day-2), and late (Day-9 for RMA, and Day-12 for RMA-KR). Results show the cytokine concentrations measured (n=3-12 mice/group).

Figure S3.

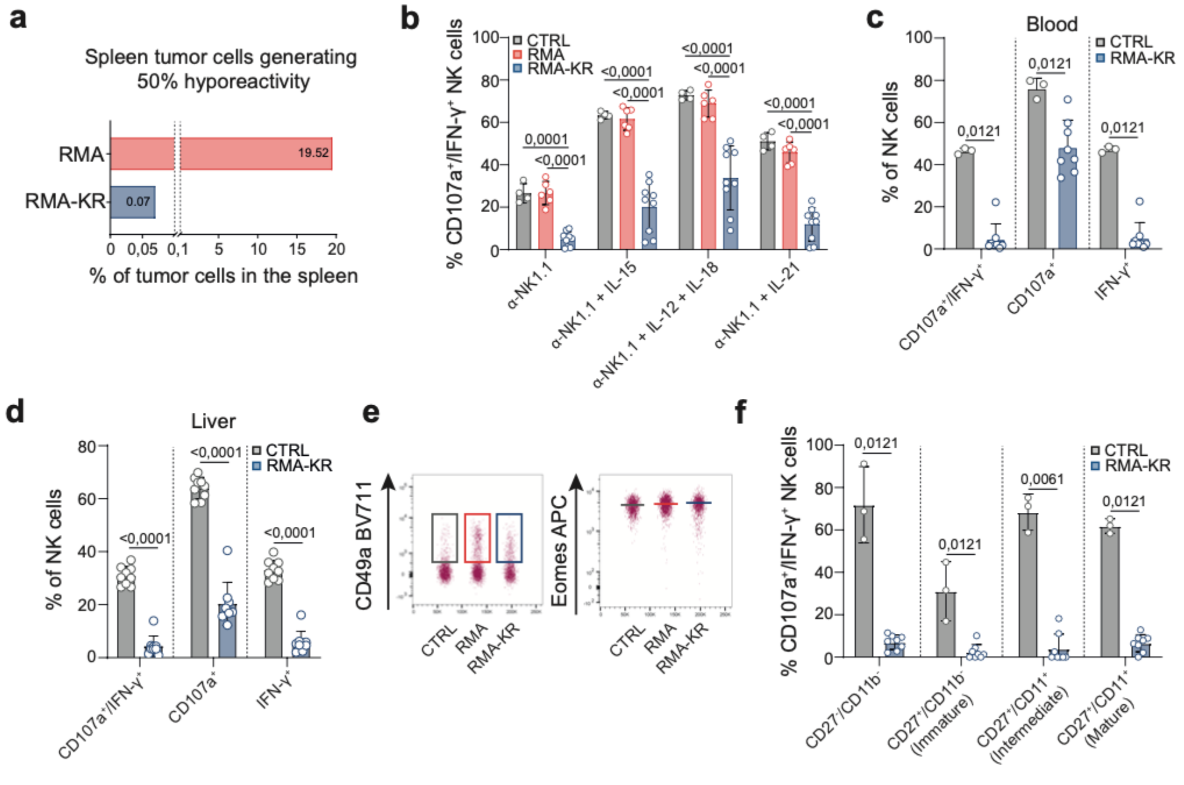

Figure S3: **RMA-KR tumor progression induces NK cell dysfunction**

(A) Frequency of tumor cells in the spleen inducing 50% decrease of CD107a+ IFN-γ+ NK cells after NK1.1 stimulation *ex vivo*, calculated based on results shown in Figure 3.

(B) Flow cytometry analysis of surface CD107 and intracellular IFN-γ in spleen NK cells from tumor-bearing mice at late stages of tumor growth. NK cells were restimulated in the indicated conditions (n=4-9 mice/group). Graphs show means ± SD and Two-way ANOVA analysis followed by Tukey's multiple comparison test was performed (p-value are presented).

(C-D) Flow cytometry analysis of surface CD107 and intracellular IFN-γ in NK cells from the indicated organs of tumor-bearing mice at late stages of tumor growth, after NK1.1-mediated stimulation (n=3-9 mice/group). Graphs show means ± SD and Mann-Whitney analysis was performed (p-value are presented).

(E) Flow cytometry analysis of CD49a and Eomes expression in spleen NK cells from the indicated mice.

(F) Flow cytometry analysis of surface CD107 and intracellular IFN-γ in the indicated spleen NK cell maturation stages at late stages of RMA-KR tumor growth or control mice, after NK1.1-mediated stimulation (n=3-8 mice/group). Graphs show means ± SD and Mann-Whitney analysis was performed (p-value are presented).

Figure S4.

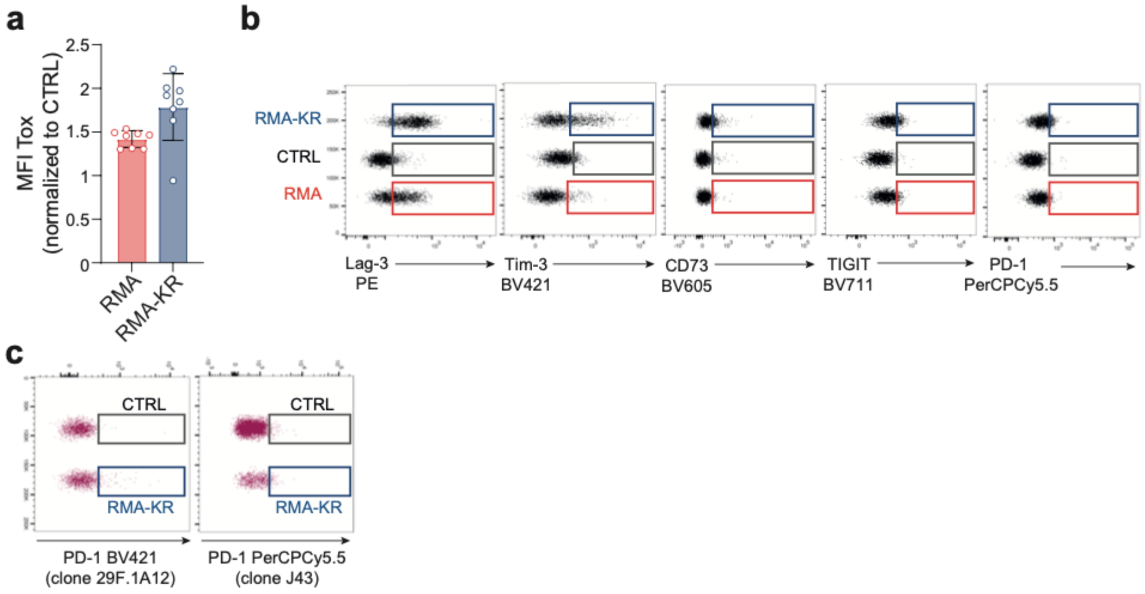

Figure S4: Expression of Tox and ICPs in dysfunctional NK cells

(A) Flow cytometry analysis of intranuclear Tox expression in NK cells from RMA- or RMA-KR bearing mice at late stages of lymphoma growth. Bar graphs show the mean fluorescence intensity of Tox staining in gated NK cells from different mice (dots), relative to control NK cells (n=8 mice/group).

(B) Representative flow cytometry dot plots showing the expression of the indicated immune checkpoints in NK cells from control or tumor-bearing mice.

(C) Flow cytometry analysis of Pd1 expression in NK cells from tumor-bearing mice using the indicated antibodies.

Figure S5.

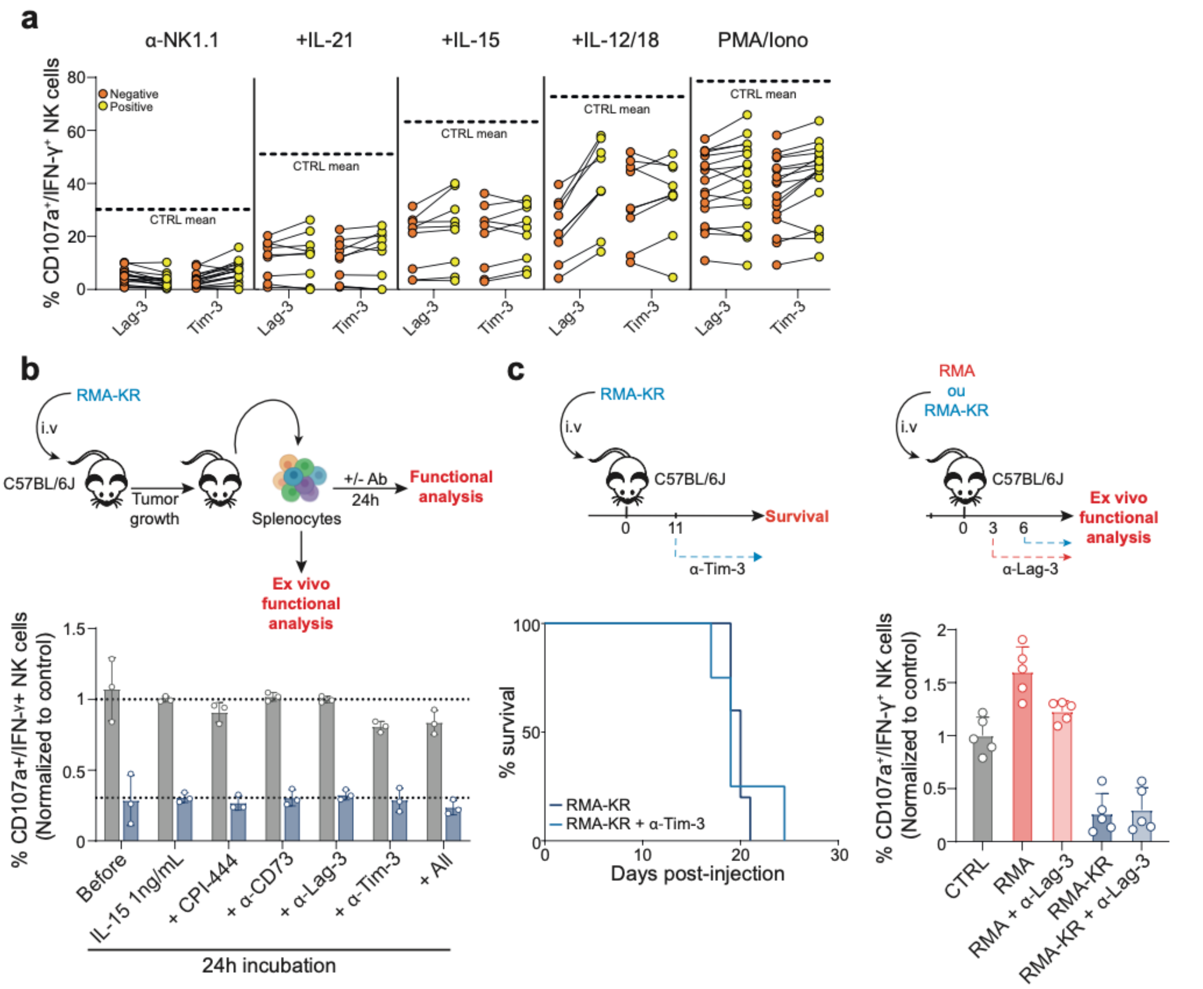

Figure S5: ICPs are not associated with NK cell dysfunction

(A) Co-expression of CD107a and IFN-γ relative to the expression of the indicated ICPs in NK cells stimulated in the indicated conditions (n=9-17 mice/group).

(B) Splenocytes from control or RMA-KR bearing mice were cultured 24h *in vitro* in the presence of combinations of blocking antibodies against ICPs, or with CPI-444, an inhibitor of Adenosine receptor A2AR, as indicated, and their reactivity to NK1.1-mediated stimulation was measured (n=3 mice/group) before or after incubation. The results are normalized to control NK cells for each culture conditions.

(C) C57BL/6 RMA or RMA-KR bearing mice were treated i.p. with Tim3 or Lag3 blocking antibodies (200ug) started at the indicated times after tumor cell injection (corresponding to their expression) and every 4 days. Mouse survival was monitored over time for Tim3 blockade (left) and NK cell reactivity was monitored using NK1.1-mediated stimulation for Lag3 blockade (right). (n=5 mice/group)

Figure S6.

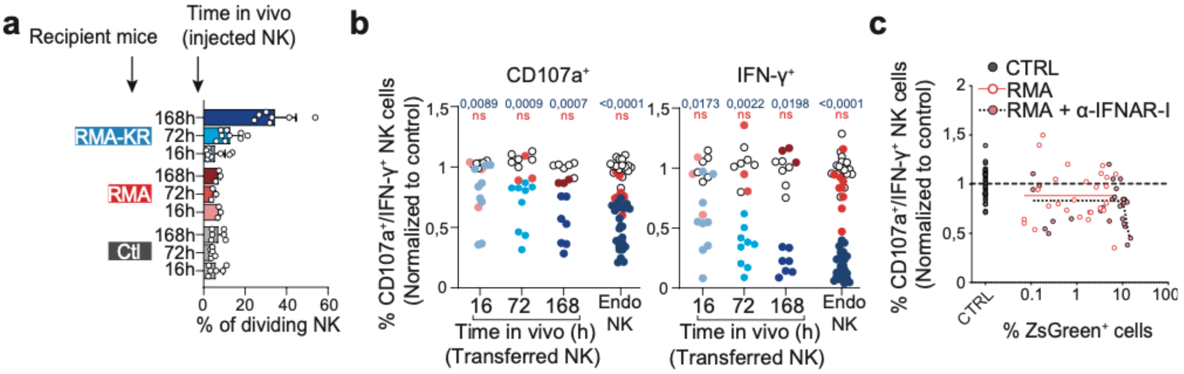

Figure S6: **Dynamics of NK cell dysfunction and proliferation in tumor-bearing mice**

(A) NK cells from Ly5a x C57BL/6 mice were enriched by negative depletion, stained with CTV and were injected 168h, 72h or 16h before end-stage of tumor growth in RMA or RMA-KR bearing C57BL/6 mice, as outlined in Figure 6A; control mice not injected with tumor were also included (N=3-9 per condition). At the indicated time point (Day 8 after RMA injection, or Day 15 after RMA-KR injection), transferred mice were sacrificed, and spleen cells were analyzed by flow cytometry. The percentage of divided transferred NK cells was monitored by the CTV dilution upon cell division. Graphs show means ± SD

(B) Reactivity of purified NK cells to NK1.1-mediated stimulation, as determined by expression of surface CD107a or intracellular IFN-γ and normalized to endogenous NK cells from Control. Graphs show the normalized percentage of CD107a+ or IFN-γ+ NK cells in individual mice are shown on the right. Each dot represents a single mouse and a Kruskal-Wallis's analysis followed by Dunn's multiple comparison test was performed (n.s P>0,05; *P<0,05; **P<0,01; ****P<0,0001).

(C) Results show NK cell reactivity in control or RMA bearing mice, previously treated or not with blocking IFNAR antibody, relative to the percentage of tumor cells in the spleen (n=27-33 mice/group). The curves were fitted using a non-linear model.

Figure S7.

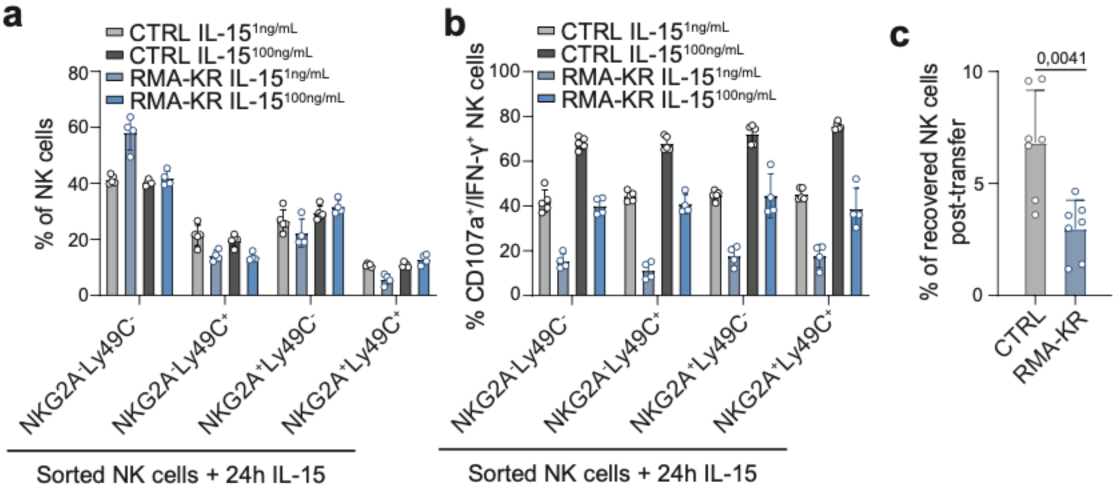

Figure S7 : **The reversion of NK cell dysfunction is independent of education process and sub-population selection**

(A-B) NK cells were sorted from RMA-KR bearing mice at late stages of tumor growth. They were then treated with IL-15 at the indicated concentration o/n before analysis. (A) Proportion of NK cells expressing education markers NKG2A and/or Ly49C and (B) reactivity of the corresponding subsets to NK1.1-mediated stimulation, as determined by expression of surface CD107a or intracellular IFN-γ by flow cytometry. Graphs show means ± SD and each dot represents a single mouse.

(C) NK cells were sorted from RMA-KR bearing mice at late stages of tumor growth or control mice and adoptively transferred into Ly5a x C57BL/6 mice. The percentage of NK cells recovered respective to the number of cells transferred was determined in the spleen of recipient mice 7 days post-transfer.

Table S1: Lists of genes differentially expressed (FC>2 and adj.p value <0.05) between RMA and control, RMA-KR and control or RMA and RMA-KR conditions.

Table S2: Functional annotation of genes differentially expressed between RMA-KR and control conditions in NK cells, as determined using Metascape. Redundant functional terms were removed to simplify the analysis.

Table S3: antibodies used in this study.
